# Enzyme-Enhanced Extraction of Phenolic Compounds and Proteins from Flaxseed Meal

**DOI:** 10.5402/2013/521067

**Published:** 2012-10-23

**Authors:** Bernardo Dias Ribeiro, Daniel Weingart Barreto, Maria Alice Zarur Coelho

**Affiliations:** School of Chemistry, Federal University of Rio de Janeiro, Ilha do Fundão, 21945-970, Rio de Janeiro, RJ, Brazil

## Abstract

Flaxseed (*Linum usitatissimum*) meal, the main byproduct of the flaxseed oil extraction process, is composed mainly of proteins, mucilage, and phenolic compounds. The extraction methods of phenolics either commonly employed the use of mixed solvents (dioxane/ethanol, water/acetone, water/methanol, and water/ethanol) or are done with the aid of alkaline, acid, or enzymatic hydrolysis. This work aimed at the study of optimal conditions for a clean process, using renewable solvents and enzymes, for the extraction of phenolics and proteins from flaxseed meal. After a screening of the most promising commercial preparations based on different carbohydrases and proteases, a central composite rotatable design and a mixture design were applied, achieving as optimal results a solution containing 6.6 and 152 g kg^−1^_meal_ of phenolics and proteins, respectively. The statistical approach used in the present study for the enzyme-enhanced extraction of phenolics and proteins from the major flaxseed byproduct was effective. By means of the sequential experimental design methodology, the extraction of such compounds was increased 10-fold and 14-fold, when compared to a conventional nonenzymatic extraction.

## 1. Introduction

**Figure 1:**
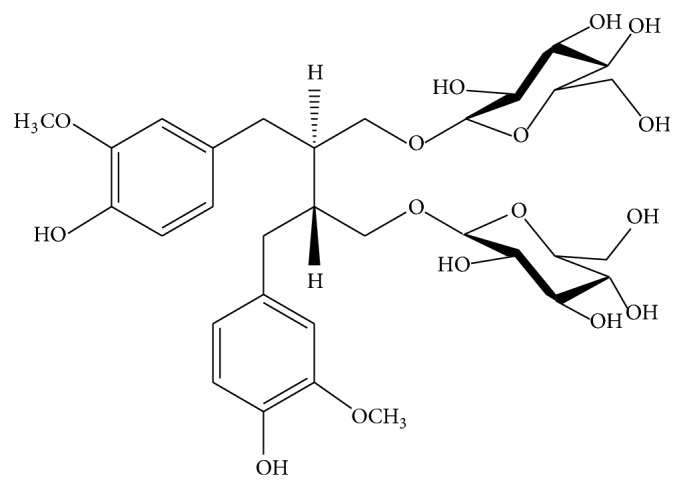
Lignan secoisolariciresinol diglucoside.

Flaxseed (*Linum usitatissimum*) meal is the main byproduct from the flaxseed oil extraction process, being primarily used as a ruminant feed. The meal is composed of three important fractions: proteins (over 300 g kg^−1^), which are rich in arginine and glutamine; amino acids that are very important in the prevention and treatment of heart diseases and in supporting the immune system; mucilage (approximate content of 80 g kg^−1^), which is a mixture of neutral arabinoxylans and rhamnogalacturonans, with good water-holding capacities and high viscosity; phenolic compounds, such as *p*-coumaric and ferulic acids, lignan secoisolariciresinol, which is presented glycosylated (Figure 1) and/or esterified with 3-hydroxy-3-methylglutaric acid to form oligomers. The content of secoisolariciresinol diglucoside in flaxseed is 2-3 g kg^−1^, and about 10–40 g kg^−1^ in defatted flaxseed powder [1–5].

In humans and animals, secoisolariciresinol is transformed by the anaerobic intestinal microflora into the mammalian lignans, enterolactone, and enterodiol, which are capable of binding at low levels to estrogen receptors. Additionally, these lignans have antioxidant, hypocholesterolemic, and antiatherosclerotic activities and inhibit the development of type 1 and type 2 diabetes, and mammary, prostatic, and colonic tumors [3, 6–9].

Lignans and total phenolic compounds are commonly extracted by using mixed solvents, such as dioxane/ethanol, water/acetone, water/methanol, and water/ethanol, followed by an ultrafiltration step for their recovery. Additionally, they can be extracted with the aid of alkaline or acid (e.g., hydrochloric acid) hydrolysis, but this method could be destructive for the target compounds whether too long heating periods or too high concentrations are used. Since flaxseed meal contains mucilage and proteins that could reduce access to the inner seed coat, enzymatic hydrolysis of these components could improve the release of phenolics and also release proteins which could aggregate value to the final product, depending on the application [3, 6, 9–12]. Furthermore, protein hydrolyzates could be applied as food additives, contributing for their functional properties [8].

Therefore, the objective of this work was the development of a clean process, based on renewable solvents and enzymes (carbohydrases and proteases), for the extraction of phenolics and proteins from flaxseed meal, by using experimental design techniques.

## 2. Materials and Methods

### 2.1. Materials

The flaxseed meal used in this work was donated by Olvepin (Indústria de Óleos Vegetais Pindorama Ltda, Panambi, Brazil). The following enzymes' preparations were obtained from Novozymes Latin America (Araucaria, Brazil) and Vetec Química Fina Ltda (Duque de Caxias, Brazil).

- Ultrazym: 3400 FDU 20°C·mL^−1^, density 1.2 g·mL^−1^, FDUs = Ferment Depectinization Units, enzyme quantity which depolymerizes 33.3 mL standard juice containing 0.4 g·L^−1^ pectin at pH 3.5, 55°C in 2 h.
- Viscozyme: 100 FBG·g^−1^, density 1.2 g·mL^−1^, FBG = Fungal Beta-Glucanase Units, enzyme quantity which hydrolyzes beta-glucan to reducing sugars corresponding to 1 *μ*mol glicose per minute at pH 5.0, 30°C during 30 minutes.
- Proteases: Neutrase, Alcalase, Flavourzyme, Papain, Pepsin, and Pancreatin.

### 2.2. Analytical Methods

The phenolic compounds content was determined according to the Folin-Denis method [13], using tannic acid as standard. Soluble proteins content was determined according to the Lowry method [14], using bovine serum albumin as standard, whereas reducing sugars content was determined by the Somogyi method [15]. For the measurement of proteolytic activity, enzyme solution was added to an azocasein solution (1 g L^−1^) in universal buffer pH 6.0, resulting in a final enzyme concentration of 0.5 g·L^−1^ (or g·kg^−1^) and maintained at 32°C for 20 min. The reaction was stopped by the addition of a trichloroacetic acid solution (200 mL L^−1^), the samples were centrifuged, and then KOH solution (280 g·L^−1^) was added to the supernatant, with the absorbance at 428 nm registered [16]. All analyses were done in triplicate using a Shimadzu UV-1800 spectrophotometer.

### 2.3. Experimental Procedure

The first step for the process optimization was the screening of the effects of different hydrolytic enzymes acting on polysaccharides and proteins, allowing more phenolics and proteins extraction. Hence, in addition to monitoring phenolics and proteins contents, reducing sugar content was also evaluated which was associated with polysaccharide hydrolysis. For this extraction, two solvent compositions were evaluated: pure (distilled) water and ethanolic solution (100 mL L^−1^). This ethanol concentration was selected as the maximum which could be evaluated for the improvement of the components extraction, with no damages to enzyme activity [17]. For this set of experiments, the fixed conditions used were meal concentration, 0.2 g mL^−1^; temperature, 50°C; time, 1.5 h; agitation speed, 200 rpm. Enzyme concentrations added in each trial were 10 mL L^−1^ for Ultrazym, Viscozyme, and Alcalase, whereas lower charges were used for Neutrase (4.1 g L^−1^), Flavourzyme (3.5 mL L^−1^), Papain (7.5 g L^−1^), Pancreatin (14.5 g L^−1^), and Pepsin (20.7 g L^−1^), according to their activities, so that, in all experiments where proteases were used, the proteolytic charge was the same. In the trials where Pepsin was used, the pH was adjusted to 3, according to the recommendation of the supplier.

Once the most promising enzyme preparations were chosen, a central composite rotatable design (CCRD) was performed, considering ethanol concentration (0–100 mL·L^−1^), meal concentration (0.05–0.20 g·mL^−1^), and enzyme concentration (0–20 mL·L^−1^) as factors. The proportion between the three enzyme preparations used in these experiments was maintained constant (1/1/1).

The optimized conditions pointed out by the CCRD were used in the following set of experiments, which was a simplex centroid mixture design, in order to determine the optimal proportion between the three enzyme preparations (0–20 mL·L^−1^ for each one) for the release of phenolic compounds. All results from experimental designs were analyzed using the software Statistica 6.0.

## 3. Results and Discussion

### 3.1. Enzyme Screening

Due to different mechanisms of action towards substrates that can be presented by proteases, such as nucleophilic attack (e.g., serine and cysteine proteases) or acid/base catalysis (e.g., aspartic or metalloproteases) [18], an enzyme screening considering several proteolytic preparations was performed in order to compare their action on flaxseed meal (Table 1). Some authors used other proteases as ficin, thermolysin, and trypsin in the processing of proteins isolated from defatted flaxseed meal reaching 73 to 99% of peptides with a size below 1 kDa [19–21].

Both pectinases-based (Ultrazym) and hemicellulases-based (Viscozyme) preparations presented a considerable action on mucilage hydrolysis, evidenced by the higher release of phenolic compounds and proteins, generally improved in the presence of ethanol. Some authors reported the use of polysaccharide-degrading enzymes, alone or in combination, to enable the utilization of flaxseed meal as animal feed, lowering in 34.7% mucilage content with combination of cellulase, mannase, and pectinases [22], while others applied these enzymes and soaking with sodium bicarbonate solutions for the improvement of extraction and recovery of flaxseed proteins [23]. The authors showed similar results regarding protein recovery (70%) when they used a seed concentration of 0.2 g mL^−1^, in a solution containing 0.01 M acetate buffer, pH 4.0 at 40°C, and the preparation Viscozyme for 3 h, and a treatment with NaHCO_3_ 0.10 M for 12 h, 30°C, using a ratio seed/solvent 1/10.

Use of cellulases was also reported, after sequential extraction with 70 mL L^−1^ methanol during 16 h followed by 0.1 M sodium hydroxide hydrolysis during 6 h, for mucilage hydrolysis and observed improved release of phenolics from flaxseed hulls. The hull concentration used was 0.025 g mL^−1^, at 40°C, for 6 h and 0.1 M citrate-phosphate buffer pH 2.8. Using these conditions, the maximum secoisolariciresinol concentration obtained was 40.75 mg·g^−1^ [9]. In another work, the authors used cellulases only for the aid of the solubilization of flaxseed meal proteins by 0.5 M NaOH [19, 21]. Whereas, another systems already have been tested without enzymes, using 700 (mL L^−1^) ethanol, at 40°C for 28 h achieved 89.75 mg g^−1^ of lignans from flaxseed meal [3], or using pressurized (5.2 MPa) low polarity water at 170°C obtained 21 mg g^−1^ of lignans, pH 9 with meal concentration of 0.01 g mL^−1^, and 225 mg g^−1^ of proteins using water at 160°C, 5.2 MPa, pH 9, and meal concentration of 0.005 g mL^−1^, during 3 to 7 h [24], or aided by nonthermal energies, as microwave and ultrasound, using 400 (mL L^−1^) ethanol for presoaking of defatted flour of flaxseed hulls with 80 W ultrasonic treatment for 5 min and 130 W microwave irradiation during 90.5 s reaching 11.7 g·kg^−1^ of secoisolariciresinol [25].

Amongst the evaluated proteolytic preparations, it was observed that ethanol inhibited slightly the action of Pancreatin, Papain, and Flavourzyme. Despite that, the content of phenolic compounds and proteins was increased in comparison with the control conditions (without enzymes), with 5.1-fold and 3.5-fold increment, respectively. Although Pancreatin promoted the best results for phenolics release, the final solutions obtained (using both solvent compositions) presented undesired organoleptic characteristics. For this reason, Alcalase, which was the second best proteolytic preparation, was selected for further experiments.

### 3.2. Central Composite Design

The preparations Alcalase, Ultrazym, and Viscozyme were used for the CCRD, which allowed the determination of optimized conditions in terms of ethanol concentration in solvent, meal concentration, and enzyme concentration. Experimental results (Table 2) were analyzed in Statistica 6.0. For each response, the statistic models were adjusted in order to present the highest coefficient of determination (*R* ^2^) with all terms statistically significant (*P* value < 0.05) [26, 27]. Then, optimized conditions were calculated by response surface methodology using the global desirability approach. For this, each one of the three response variables was converted to individual desirability functions (which varies from 0 to 1), and then the global desirability function (objective function) was calculated as a geometric mean of all individual desirability functions [26].

The relevant terms were all three linear terms of factors, quadratic term of the ratio meal/solvent, and interaction between linear terms of ethanol concentration and ratio meal/solvent with adjusted *R* ^2^ of 0.613, 0.937, and 0.883 for phenolic compounds, proteins, and reducing sugars, respectively. The multiresponse optimization was programmed with focus on the maximization of the phenolic compounds' extraction, so that the other response variables were maintained at intermediate values. With this scenario, the global optimal conditions were pure water as solvent (ethanol concentration equal to 0 mL L^−1^); meal concentration, 0.152 g mL^−1^; total enzyme concentration (the sum of the three preparations, equally added), 20 mL L^−1^. These conditions obtained were not surprising since at Table 1 can be verified, in presence of 100 mL·L^−1^ ethanol, the extraction of phenolic compounds had been decreased when using Viscozyme and Alcalase, and with a high enzyme content, the proteins and polysaccharides were faster degraded, and more phenolic compounds released from flaxseed meal.

### 3.3. Mixture Design

**Figure 2:**
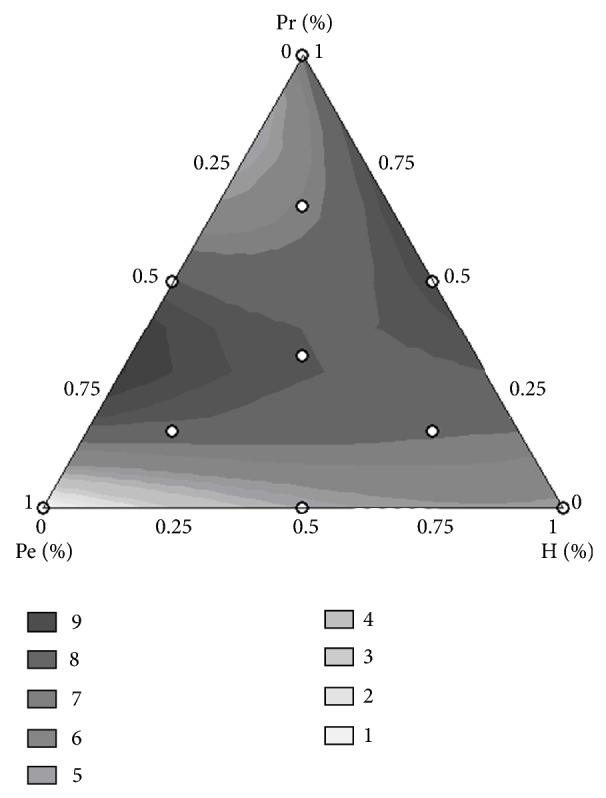
Response surface to the enzyme formulation for optimization of enzymatic extraction of phenolics. Pe: pectinases (Ultrazym), H: hemicellulase (Viscozyme), Pr: protease (Alcalase).

The optimized conditions pointed out by the desirability approach were considered for the mixture design, where different proportions between the preparations Ultrazym (Pe, pectinase), Viscozyme (H, hemicellulase), and Alcalase (Pr, protease) were investigated (Table 3). Using the modified full cubic model, the parameters' coefficients which were statistically significant were linear terms of hemicellulase and protease, and the interaction between pectinase and protease with adjusted *R* ^2^ 0.761. Focusing on maximizing the phenolic compounds content (mg·g^−1^), the optimized enzyme formulation for use in phenolics extraction from flaxseed meal should contain 6.9 mL L^−1^ of Ultrazym and 3.1 mL L^−1^ of Alcalase (Figure 2). Statistical analysis indicated that, with this optimized conditions, hemicellulases (present in the preparation Viscozyme) were not necessary. Phenolics, proteins, and reducing sugars contents predicted in the optimized conditions were 6.6, 152, and 25.4 mg g^−1^, respectively.

When compared to the enzyme charges used in the former design, the volumes of both enzyme preparations were reduced, without significant loss in the recovery of the target compounds. Therefore, the use of a mixture design subsequently to a CCRD showed important improvement in the process for extraction of phenolics and proteins from flaxseed meal.

## 4. Conclusion

The use of several commercial enzyme preparations for the extraction of phenolics and proteins from the major flaxseed byproduct, flaxseed meal, was investigated. Two carbohydrase-based preparations (Ultrazym and Viscozyme) and an alkaline protease-based preparation (Alcalase) were used in a central composite rotatable design, where the optimal conditions pointed out were meal concentration of 0.152 g mL^−1^ and total enzyme concentration of 20 mL L^−1^, in a system containing only pure (distilled) water as solvent. When evaluated in a simplex centroid mixture design, the optimal charges of the enzymes were 6.9 mL L^−1^ for Ultrazym and 3.1 mL L^−1^ for Alcalase, thus reduced as compared to the former optimization. At the end of the sequential optimization, the concentrations of phenolic compounds and proteins in the hydrolyzates were increased 10-fold and 14-fold, respectively, in comparison to the control (without the addition of enzymes) experiment. The process proposed in the present study is a promising and alternative technology for the recovery of valuable components from an agricultural byproduct, meeting sustainability criteria due the use of a green solvent and renewable catalysts.

## Figures and Tables

**Table 1:** Enzyme screening for hydrolysis of flaxseed meal.

	Water (mL·L^−1^)	EtOH (mL·L^−1^)	Enzyme	(g·L^−1^ or mL·L^−1^)	Phenolics (mg·g^−1^)	Proteins (mg·g^−1^)	Sugars (mg·g^−1^)
1	1000	0	—	0	0.90 ± 0.05	11.73 ± 0.37	0.51 ± 0.05
2	900	100	—	0	0.83 ± 0.04	9.64 ± 0.30	0.65 ± 0.05
3	1000	0	Ultrazym	10	4.27 ± 0.09	53.94 ± 0.45	23.27 ± 0.30
4	900	100	Ultrazym	10	4.59 ± 0.08	67.14 ± 0.51	24.53 ± 0.35
5	1000	0	Viscozyme	10	6.14 ± 0.10	54.18 ± 0.42	25.31 ± 0.38
6	900	100	Viscozyme	10	4.46 ± 0.08	67.79 ± 0.53	23.96 ± 0.46
7	1000	0	Neutrase	4.1	1.75 ± 0.07	29.55 ± 0.45	2.49 ± 0.06
8	900	100	Neutrase	4.1	1.98 ± 0.06	33.19 ± 0.41	4.00 ± 0.10
9	1000	0	Alcalase	10	2.99 ± 0.09	32.47 ± 0.42	3.15 ± 0.08
10	900	100	Alcalase	10	2.47 ± 0.05	36.09 ± 0.54	2.39 ± 0.04
11	1000	0	Flavourzyme	3.5	1.78 ± 0.04	30.21 ± 0.21	5.08 ± 0.06
12	900	100	Flavourzyme	3.5	1.56 ± 0.06	22.94 ± 0.25	9.43 ± 0.19
13	1000	0	Papain	7.5	2.13 ± 0.07	27.95 ± 0.35	3.52 ± 0.07
14	900	100	Papain	7.5	1.26 ± 0.04	21.64 ± 0.38	6.30 ± 0.12
15	1000	0	Pepsin	20.7	1.97 ± 0.06	32.21 ± 0.42	5.57 ± 0.11
16	900	100	Pepsin	20.7	2.79 ± 0.08	36.45 ± 0.57	9.91 ± 0.16
17	1000	0	Pancreatin	14.5	4.62 ± 0.08	41.04 ± 0.70	7.27 ± 0.12
18	900	100	Pancreatin	14.5	3.63 ± 0.09	30.62 ± 0.55	9.50 ± 0.14

**Table 2:** Optimization of flaxseed meal hydrolysis by central composite design.

Assays	EtOH (mL·L^−1^)	Meal/solvent (g·mL^−1^)	Enzyme (mL·L^−1^)	Phenolics (mg·g^−1^)	Proteins (mg·g^−1^)	Sugars (mg·g^−1^)
1	20	0.080	4	5.52	186.97	56.28
2	20	0.080	16	5.46	235.46	79.48
3	20	0.170	4	3.82	102.27	25.85
4	20	0.170	16	5.77	118.86	28.16
5	80	0.080	4	6.44	180.77	61.03
6	80	0.080	16	7.55	221.66	66.94
7	80	0.170	4	0.80	30.62	9.16
8	80	0.170	16	4.22	78.04	19.52
9	0	0.125	10	8.06	141.65	39.79
10	100	0.125	10	3.69	105.22	35.26
11	50	0.050	10	1.61	298.40	83.32
12	50	0.200	10	1.75	47.41	15.01
13	50	0.125	0	0.50	41.08	4.56
14	50	0.125	20	9.75	171.04	41.07
15 (C)	50	0.125	10	6.01	136.67	39.53
16 (C)	50	0.125	10	5.93	137.85	38.02
17 (C)	50	0.125	10	6.25	141.05	39.74

**Table 3:** Optimization of enzyme ratio in the flaxseed meal hydrolysis by mixture design.

Assays	% Pe	% H	% Pr	Phenolics (mg·g^−1^)	Proteins (mg·g^−1^)	Sugars (mg·g^−1^)
1	1.00	0.00	0.00	0.55	115.69	37.14
2	0.00	1.00	0.00	5.30	179.01	44.50
3	0.00	0.00	1.00	7.10	114.52	11.90
4	0.50	0.50	0.00	5.35	161.72	43.70
5	0.50	0.00	0.50	8.87	159.00	31.40
6	0.00	0.50	0.50	9.56	164.75	41.43
7	0.67	0.17	0.17	6.72	158.73	43.61
8	0.17	0.67	0.17	6.17	149.25	37.25
9	0.17	0.17	0.67	5.72	144.25	34.16
10	0.33	0.33	0.33	8.45	175.50	41.16

Pe: pectinases (Ultrazym).

H: hemicellulase (Viscozyme).

Pr: protease (Alcalase).

## References

[1] Oomah B. D. (2001). Flaxseed as a functional food source. *Journal of the Science of Food and Agriculture*.

[2] Warrand J., Michaud P., Picton L. (2005). Structural investigations of the neutral polysaccharide of *Linum usitatissimum* L. seeds mucilage. *International Journal of Biological Macromolecules*.

[3] Zhang Z. S., Li D., Wang L. J. (2007). Optimization of ethanol-water extraction of lignans from flaxseed. *Separation and Purification Technology*.

[4] Naran R., Chen G., Carpita N. C. (2008). Novel rhamnogalacturonan I and arabinoxylan polysaccharides of flax seed mucilage. *Plant Physiology*.

[5] Xu Y., Hall C., Wolf-Hall C. (2008). Antifungal activity stability of flaxseed protein extract using response surface methodology. *Journal of Food Science*.

[6] Eliasson C., Kamal-Eldin A., Andersson R., Åman P. (2003). High-performance liquid chromatographic analysis of secoisolariciresinol diglucoside and hydroxycinnamic acid glucosides in flaxseed by alkaline extraction. *Journal of Chromatography A*.

[7] Zhang W., Wang X., Liu Y. (2008). Dietary flaxseed lignan extract lowers plasma cholesterol and glucose concentrations in hypercholesterolaemic subjects. *British Journal of Nutrition*.

[8] Mueller K., Eisner P., Yoshie-Stark Y., Nakada R., Kirchhoff E. (2010). Functional properties and chemical composition of fractionated brown and yellow linseed meal (*Linum usitatissimum* L.). *Journal of Food Engineering*.

[9] Renouard S., Hano C., Corbin C. (2010). Cellulase-assisted release of secoisolariciresinol from extracts of flax (*Linum usitatissimum*) hulls and whole seeds. *Food Chemistry*.

[10] Westcott N. D., Paton D. (2000). A complex containing lignan, phenolic and aliphatic substances from flax and process for preparing. *WO Patent*.

[11] Dobbins T. A., Wiley D. B. (2004). Process for recovering secoisolariciresinol diglycoside from defatted flaxseed. *US Patent*.

[12] Puri M., Sharma D., Barrow C. J. (2011). Enzyme-assisted extraction of bioactives from plants. *Trends in Biotechnology*.

[13] Waterman P. G., Mole S. (1994). *Analysis of Phenolic Plant Metabolites*.

[14] Lowry O. H., Rosebrough N. J., Farr A. L., Randall R. J. (1951). Protein measurement with the Folin phenol reagent. *The Journal of biological chemistry*.

[15] Somogyi M. (1952). Notes on sugar determination. *The Journal of Biological Chemistry*.

[16] Amaral P. F. F. (2010). *Produção de Lipase de Yarrowia Lipolytica em Biorreator Multifásico*.

[17] Chen H., Jin S. (2006). Effect of ethanol and yeast on cellulase activity and hydrolysis of crystalline cellulose. *Enzyme and Microbial Technology*.

[18] Beynon R., Bond J. S. (2001). *Proteolytic Enzymes, a Practical Approach*.

[19] Udenigwe C. C., Lin Y. S., Hou W. C., Aluko R. E. (2009). Kinetics of the inhibition of renin and angiotensin I-converting enzyme by flaxseed protein hydrolysate fractions. *Journal of Functional Foods*.

[20] Udenigwe C. C., Adebiyi A. P., Doyen A., Li H., Bazinet L., Aluko R. (2012). Low molecular weight flaxseed protein-derived arginine-containing peptides reduced blood pressure of spontaneously hypertensive rats faster than amino acid form of arginine and native flaxseed protein. *Food Chemistry*.

[21] Udenigwe C. C., Lu Y. L., Han C. H., Hou W. C., Aluko R. E. (2009). Flaxseed protein-derived peptide fractions: antioxidant properties and inhibition of lipopolysaccharide-induced nitric oxide production in murine macrophages. *Food Chemistry*.

[22] Slominski B. A., Meng X., Campbell L. D., Guenter W., Jones O. (2006). The use of enzyme technology for improved energy utilization from full-fat oilseeds. Part II: flaxseed. *Poultry Science*.

[23] Wanasundara P. K. J. P. D., Shahidi F. (1997). Removal of flaxseed mucilage by chemical and enzymatic treatments. *Food Chemistry*.

[24] Ho C. H. L., Cacace J. E., Mazza G. (2007). Extraction of lignans, proteins and carbohydrates from flaxseed meal with pressurized low polarity water. *LWT—Food Science and Technology*.

[25] Zhang W., Xu S. (2007). Microwave-assisted extraction of secoisolariciresinol diglucoside from flaxseed hull. *Journal of the Science of Food and Agriculture*.

[26] Calado V., Montgomery D. C. (2003). *Planejamento de Experimentos Usando o Statistica*.

[27] Rodrigues M. I., Iemma A. F. (2005). *Planejamento De Experimentos e Otimização De Processos*.

